# Health status of the advanced elderly in six european countries: results from a representative survey using EQ-5D and SF-12

**DOI:** 10.1186/1477-7525-8-143

**Published:** 2010-11-29

**Authors:** Hans-Helmut König, Dirk Heider, Thomas Lehnert, Steffi G Riedel-Heller, Matthias C Angermeyer, Herbert Matschinger, Gemma Vilagut, Ronny Bruffaerts, Josep M Haro, Giovanni de Girolamo, Ron de Graaf, Viviane Kovess, Jordi Alonso

**Affiliations:** 1 Department of Medical Sociology and Health Economics, University Medical Center Hamburg-Eppendorf, Martinistr. 52, D-20246 Hamburg, Germany; 2 Department of Social Medicine, University of Leipzig, Philipp-Rosenthal-Str. 55, D-04103 Leipzig, Germany; 3 Center for Public Mental Health, Untere Zeile 13, A-3482 Gösing am Wagram, Austria; 4 Department of Psychiatry, University of Leipzig, Semmelweisstr. 10, D-04103 Leipzig, Germany; 5 Health Services Research Unit, Institut Municipal d'Investigació Mèdica (IMIM) & CIBER en Espidemiología y Salud Pública (CIBERESP), Carrer del Doctor Aiguader, 88, Edifici PRBB, E-08003 Barcelona, Spain; 6 Department of Neurosciences, Katholieke Universiteit Leuven, UZ Herestraat 49 - bus 7003, B-3000 Leuven, Belgium; 7 Sant Joan de Déu-SSM. Fundació Sant Joan de Déu Santa Rosa 39-57, E-08950, Barcelona, Spain; 8 Department of Mental Health, AUSL di Bologna, Viale Pepoli 5, I-40123 Bologna, Italy; 9 Netherlands Institute of Mental Health and Addiction, Da Costakade 45, NL-3521 VS Utrecht, the Netherlands; 10 Department of Epidemiology, School for Public Health (EHESP), Paris Descartes University, 12 Av De Lamballe, F-75016 Paris 16, France

## Abstract

**Background:**

Due to demographic change, the advanced elderly represent the fastest growing population group in Europe. Health problems tend to be frequent and increasing with age within this cohort.

**Aims of the study:**

To describe and compare health status of the elderly population in six European countries and to analyze the impact of socio-demographic variables on health.

**Methods:**

In the European Study of the Epidemiology of Mental Disorders (ESEMeD), representative non-institutionalized population samples completed the EQ-5D and Short Form-12 (SF-12) questionnaires as part of personal computer-based home interviews in 2001-2003. This study is based on a subsample of 1659 respondents aged ≥ 75 years from Belgium (n = 194), France (n = 168), Germany (n = 244), Italy (n = 317), the Netherlands (n = 164) and Spain (n = 572). Descriptive statistics, bivariate- (chi-square tests) and multivariate methods (linear regressions) were used to examine differences in population health.

**Results:**

68.8% of respondents reported problems in one or more EQ-5D dimensions, most frequently pain/discomfort (55.2%), followed by mobility (50.0%), usual activities (36.6%), self-care (18.1%) and anxiety/depression (11.6%). The proportion of respondents reporting any problems increased significantly with age in bivariate analyses (age 75-79: 65.4%; age 80-84: 69.2%; age ≥ 85: 81.1%) and differed between countries, ranging from 58.7% in the Netherlands to 72.3% in Italy. The mean EQ VAS score was 61.9, decreasing with age (age 75-79: 64.1; age 80-84: 59.8; age ≥ 85: 56.7) and ranging from 60.0 in Italy to 72.9 in the Netherlands. SF-12 derived Physical Component Summary (PCS) and Mental Component Summary (MCS) scores varied little by age and country. Age and low educational level were associated with lower EQ VAS and PCS scores. After controlling for socio-demographic variables and reported EQ-5D health states, mean EQ VAS scores were significantly higher in the Netherlands and Belgium, and lower in Germany than the grand mean.

**Conclusions:**

More than two thirds of the advanced elderly report impairment of health status. Impairment increases rapidly with age but differs considerably between countries. In all countries, health status is significantly associated with socio-demographic variables.

## Background

In 2050 about 30% of the European population will be aged ≥ 65. Due to the ongoing increase in life expectancy, about 11% will be aged ≥ 80, which constitutes a near threefold increase for this group since 2000 [1]. Therefore, the old-old are the fastest growing population group in Europe. Because elder persons have more chronic conditions and induce higher per capita health care costs [2], evaluation of health care for this population segment is of great importance.

Evaluation of health care requires the measurement of health status. During the past decades several generic and disease specific measures of health status have been developed [3]. Unlike disease specific measures, generic measures are designed to record key aspects of health status independent of diagnostic category and disease severity [4]. Thus, generic instruments may be used to compare health status of patient groups across different diseases as well as different populations, thereby providing information particularly useful to support health policy decisions [5].

Two widely used generic health-related quality of life measures are the EQ-5D and the 12 item Short Form health survey (SF-12). The EQ-5D is a simple measure to subjectively describe and value health status [6-8] . It has been used to measure and compare the health status of general population samples in various countries [9-15]. The EQ-5D consists of two parts, the EQ-5D descriptive system and the visual analogue scale (EQ-VAS). The SF-12 is a downsized version of the SF-36, which similarly allows the derivation of a summary score for physical- and mental health, respectively [16,17].

The aim of this study was to describe and compare general population health status of elderly non-institutionalized Europeans aged ≥ 75 measured by the EQ-5D and SF-12, and to study its association with socio-demographic variables. In difference to many previous cross-country comparisons, which were complicated due to the time distance of individual country surveys, differences in sampling procedures, differences in the socio-demographic variables collected, as well as possible order effects of questionnaires, this study was based on an international survey that used an identical sampling procedure, applied the same set of questionnaires and was conducted almost simultaneously in the countries compared.

## Methods

### Subjects and study design

The European Study of the Epidemiology of Mental Disorders (ESEMeD) is a cross-sectional survey conducted to investigate the prevalence of mental disorders and their effects on health status and health service use of noninstitutionalized adults (aged ≥ 18) in 6 European countries, i.e. Belgium, France, Germany, Italy, the Netherlands, and Spain. The sample is based on a stratified, multistage, cluster area probability design. More detailed information of the survey methodology and sample characteristics can be found elsewhere [15]. Individuals were interviewed in person at their homes from January 2001 to August 2003, using computer-assisted interview-techniques. The overall response rate in the six countries investigated was 61.2%, with the highest rates in Spain (78.6%) and Italy (71.2%), and lowest rates in Germany (57.8%), the Netherlands (56.4%), Belgium (50.6%) and France (45.9%). The total sample contains information from 21,425 respondents of whom 1,685 are aged ≥ 75 years. 1,659 (98.5%) of all respondents aged ≥ 75 years provided complete EQ-5D and SF-12 information; the analysis presented here is based on this number. The sample sizes of the individual countries are presented in Table 1.

**Table 1:** Sociodemographic characteristics of respondents, by country

	Total sample n = 1659	Belgium n = 194	France n = 168	Germany n = 244	Italy n = 317	Netherlands n = 164	Spain n = 572	p-value
Age								
mean (SD)	79.8 (4.3)	79.5 (4.1)	79.8 (4.6)	79.3 (4.0)	79.9 (4.7)	79.8 (4.2)	80.1 (4.3)	
median (range)	79 (75 - 100)	79 (75 - 92)	78 (75 - 96)	78 (75 - 93)	79 (75 - 100)	79 (75 - 95)	79 (75 - 98)	
weighted mean (SE)	79.7 (0.14)	79.4 (0.33)	79.5 (0.36)	79.3 (0.28)	80.1 (0.30)	79.5 (0.34)	80.3 (0.23)	0.0579^b^
Gender: n (%)^a^								
male	679 (35.1)	87 (40.6)	67 (36.7)	95 (31.8)	133 (34.8)	55 (35.2)	242 (39.0)	
female	980 (64.9)	107 (59.4)	101 (63.3)	149 (68.2)	184 (65.2)	109 (64.8)	330 (61.0)	0.4627^c^
Living arrangement: n (%)^a^								
living with partner	849 (50.7)	113 (60.8)	79 (52.4)	124 (48.3)	176 (50.5)	65 (46.9)	292 (52.8)	
living without partner	810 (49.3)	81 (39.2)	89 (47.6)	120 (51.7)	141 (49.5)	99 (53.1)	280 (47.2)	0.2048^c^
Education: n (%)^a^								
≤ 12 years	1448 (86.9)	166 (83.0)	134 (79.3)	217 (89.8)	275 (87.1)	129 (80.9)	527 (91.9)	
> 12 years	211 (13.1)	28 (17.0)	34 (20.7)	27 (10.2)	42 (12.9)	35 (19.1)	45 (8.1)	0.0003 ^c^
Employment: n (%)^a^								
paid employment	77 (5.7)	9 (4.8)	7 (4.8)	4 (1.6)	45 (14.4)	2 (1.1)	10 (2.4)	
no paid employment	1582 (94.3)	185 (95.2)	161 (95.2)	240 (98.4)	272 (85.6)	162 (98.9)	562 (97.6)	< 0.0001^c^
Household income								
mean (SD)	16512 (17148)	15763 (12450)	22999 (27094)	22193 (19152)	19183 (16683)	19306 (21125)	10155 (8729)	
median (range)	11899 (0 - 182329)	12444 (509 - 88597)	15710 (473 - 165164)	15722 (486 - 142856)	14254 (465 - 118992)	12865 (0 - 182329)	7167 (481 - 71370)	
weighted mean (SE)	19244 (588)	17010 (1093)	22613 (1986)	22191 (1228)	18294 (720)	19483 (1556)	11055 (462)	< 0.0001^b ^

^a ^Unweighted n, weighted proportion; ^b ^weighted regression analysis testing for deviation of country specific means from grand mean; ^c ^weighted chi-square test for differences between countries.

### EQ-5D

The EQ-5D questionnaire consists of five questions (items), which are related to problems in the dimensions mobility, self-care, usual activities, pain/discomfort, and anxiety/depression [6,7]. For each question, three ordinal-scaled answer categories exist which are coded as follows: 1. no problems, 2. moderate problems, 3. extreme problems. This part of the EQ-5D questionnaire is referred to as the EQ-5D descriptive system. In addition respondents are asked to value their own health state on a visual analogue scale (EQ-VAS). The EQ-VAS records a respondent's self rated valuation of health status on a scale ranging from 0 (worst imaginable health state) to 100 (best imaginable health state), providing the so-called EQ-VAS score.

### SF-12

The SF-12 is a downsized version of the 36 short form health survey (SF-36), in which a subset of 12 items/questions (of the original 36 contained within the SF-36) are used to derive a summary score for physical health (PCS score) and one for mental health (MCS score), respectively [16]. By covering the same dimensions as the SF-36, i.e. physical functioning (2 questions), role-physical functioning (2 questions), bodily pain (1 question), general health (1 question), vitality (1 question), social functioning (1 question), role-emotional functioning (2 questions), and mental health (2 questions), while using only one-third of the items, the SF-12 is able to produce the two summary scores originally developed for the SF-36 with remarkable accuracy but far less respondent burden [17]. Both the PCS- and MCS summary score are not preference-based, but have a psychometrical foundation. The scores are standardized to population norms (based on a US norm-sample), with the mean score set at 50 (SD = 10); lower scores indicate worse-, and higher scores better health. The SF-12 has been validated for use in the USA, UK and many further European countries [18].

### Socio-demographic variables

The following sociodemographic variables were used for the statistical analyses: age, gender, living arrangement (married/living with some vs. single/divorced/separated/widowed), years of education (≤ 12 vs. > 12 years), employment status (paid employment vs. no paid employment), and household income (≤ national median vs. > national median). Thus, all sociodemographic variables except for age were dichotomized: In cross-cultural comparison there is always a trade-off between the amount (or loss) of information and the amount of error (or reliability). Particularly income quite frequently is reported unreliably since respondents often do not want to declare their actual income precisely. So dichotomising this unreliable and considerably biased information results in a more correct predictor (low, high), even though less informative predictor. Educational systems in Europe considerably differ from one another, but the information "low" and "high" still holds for all the countries similarly. Therefore, dichotomizing sociodemographic variables resulted in a loss of information on the one hand which is offset by an increase in reliability on the other [19].

Household income was calculated as the sum of the respondent's own earned income, earned income of other persons living in the household, social security income, government assistance and other sources of income such as investments, alimony, etc. Missing values of the summands were imputed by taking into account age, gender, years of education, employment status and the number of persons living in the household. About 20% of the individuals had missing values in any of the summands. Missing data were imputed by means of conditional median imputation to deal with the skewness of the household income and because it is known to be robust against outliers. Therefore missing data were replaced by the median of cell groups which were defined by the categories of the age, gender, education, employment status and the number of persons living in the household. Yet, since residual (error) variance is not accounted for by conditional median imputation the estimated variance may be underestimated. Approximately 11% of individuals had missing values in the employment variable that were imputed taking into account the age, gender, marital status, years of education and income. The rest of socio-demographic variables used had less than 1% of missing values which were also imputed.

### Statistical analysis

We calculated the proportion of respondents reporting no problems, moderate problems, and extreme problems in each of the EQ-5D dimensions. Furthermore, median and mean EQ-VAS, PCS and MCS scores were calculated. These were performed for the total sample, and separately for each country and for each of the age groups (75-79, 80-84, 85+). To account for the known probability of selection into the sample, and to restore the distribution of the population within each country, estimated proportions and means were weighted. Estimates for the total sample were weighted to re-establish the relative dimension of the population across countries. Chi-square tests were employed to explore differences in proportions across countries and age-groups. To test for deviation of country and age-group specific means from the grand mean, regression analyses and effect coding for countries were used.

The effect of socio-demographic variables (age, gender, years of education, employment status, household income, living arrangement) and country on the frequency of problems in each of the EQ-5D dimensions was analysed using *bivariate *(chi-square test and simple logistic regression for the effect of age) as well as *multivariate *approaches (multiple logistic regression). The levels 'moderate problems' and 'extreme problems' were combined into one category, since the number of respondents reporting 'extreme problems' was small for most EQ-5D dimensions. Thus, the response to EQ-5D dimensions could be treated as a binary variable for logistic regression analysis. Dummy variables were created for binary independent variables and effect coding was used for the country variables. The impact of the independent variables on the EQ VAS-, PCS-, MCS scores were assessed through multiple linear regressions.

Besides weighting, inferential statistical analyses moreover took into account the clustering and stratification of the sample survey data as often done in complex survey designs. Calculations were preformed by using the software SAS (SAS Institute Inc., Cary, North Carolina, USA, Version 9.1). The level of statistical significance was set at α = 0.01.

### Ethics

Ethics committees in each participating country approved the survey procedures (Belgium: Ethics Committee of the Federal Institute of Public Health; France: Committee of the CNIL - Commission Nationale Informatique et Libertés; Germany: Ethics Committee of the University of Leipzig; Italy: Italian National Institute of Health; Netherlands: Ethics Committee of the Netherlands Institute of Mental Health and Addiction; Spain: Ethical Committee of Sant Joan de Déu Serveis de Salut Mental, and Ethical Committee of Institut Municipal d'Investigació Mèdica). An informed consent was obtained from all respondents after having been informed about the aims of the study.

## Results

### Socio-demographic sample characteristics

Of the 1,659 respondents in the total sample, 64.9% were female, 50.7% were living with a partner, and the weighted mean age was 79.7 years (Table 1). Whereas the country samples did not significantly differ with respect to these attributes, they did regarding education, employment status and household income. Of the total sample, the majority of 86.9% had 12 years of education or less (ranging from 79.3% in France to 91.9% in Spain, p = 0.0003), 5.7% of all respondents indicated paid employment (ranging from 1.1% in the Netherlands to 14.4% in Italy, p < 0.0001), and the weighted mean (net) household income was 19,244 EUR (ranging from 11,055 EUR in Spain to 22,613 EUR in France, p < 0.0001).

### Descriptive statistics and *bivariate *analysis

68.8% of all respondents reported problems in at least one of the EQ-5D dimension, ranging from 58.7% in the Netherlands to 72.3% in Italy (p = 0.0006). Moderate problems in at least one of the EQ-5D dimensions were reported by 60.0% of all respondents, ranging from 47.9% in the Netherlands to 65.9% in France, while 8.7% of the total sample indicated extreme problems in at least one of the dimensions, ranging from 6.2% in France to 13.2% in Belgium. The dimensions most frequently reported to cause problems within the total sample were pain/discomfort (55.2%), followed by mobility (50.0%), usual activities (36.6%), self-care (18.1%), and anxiety/depression (11.6%). The same ranking applies to the country samples, with the exception of Germany and Spain, where mobility caused the most problems, followed by pain/discomfort (Table 2). The country samples significantly differed regarding the proportion of respondents reporting problems in the EQ-5D dimensions usual activities (p = 0.0003), pain/discomfort (p < 0.0001), and anxiety/depression (p = 0.0004).

**Table 2:** Problems in EQ-5D dimensions, EQ VAS, PCS and MCS scores, by country

	Total sample	Belgium	France	Germany	Italy	Netherlands	Spain	p-value
*EQ-5D dimension*								
Mobility n (%)^a^								
no problems	906 (50.0)	124 (57.1)	91 (52.8)	114 (46.0)	155 (47.5)	105 (63.5)	317 (53.3)	
moderate problems	734 (48.9)	68 (41.8)	76 (46.0)	129 (53.6)	156 (50.4)	58 (35.9)	247 (45.8)	
extreme problems	19 (1.1)	2 (1.1)	1 (1.2)	1 (0.5)	6 (2.0)	1 (0.6)	8 (0.8)	0.0228^b^
Self care n (%)^a^								
no problems	1384 (81.9)	161 (82.9)	140 (83.3)	206 (84.1)	251 (77.0)	145 (88.3)	481 (81.8)	
moderate problems	239 (15.8)	27 (14.4)	25 (13.6)	35 (14.7)	57 (19.9)	17 '(10.8)	78 (15.7)	
extreme problems	36 (2.3)	6 (2.8)	3 (3.1)	3 (1.2)	9 (3.1)	2 (0.9)	13 (2.5)	0.2345^b^
Usual activities n (%)^a^								
no problems	1075 (63.4)	127 (64.2)	112 (67.4)	162 (66.1)	181 (55.1)	120 (74.0)	373 (62.4)	
moderate problems	496 (32.1)	51 (26.9)	52 (29.0)	78 (32.2)	116 (38.0)	41 23.2)	158 (29.5)	
extreme problems	88 (4.5)	16 (9.0)	4 (3.6)	4 (1.7)	20 (6.9)	3 (2.8)	41 (7.1)	0.0003^b^
Pain/discomfort n (%)^a^								
no problems	828 (44.8)	105 (50.2)	66 (38.0)	116 (47.7)	126 (37.0)	86 (51.5)	329 (55.7)	
moderate problems	742 (50.0)	80 (44.2)	94 (57.0)	117 (47.7)	174 (57.2)	65 (39.7)	212 (39.7)	
extreme problems	89 (5.2)	9 (5.6)	8 (5.1)	11 (4.5)	17 (5.9)	13 (8.8)	31 (4.6)	< 0.0001^b^
Anxiety/depression n (%)^a^								
no problems	1484 (88.4)	182 (94.0)	145 (84.6)	229 (93.3)	267 (83.1)	158 (96.4)	503 (87.4)	
moderate problems	157 (10.7)	11 (5.2)	21 (14.3)	14 (6.2)	46 (15.7)	4 (2.6)	61 (11.6)	
extreme problems	18 (0.9)	1 (0.8)	2 (1.1)	1 (0.5)	4 (1.2)	2 (1.0)	8 (1.0)	0.0004^b^
Any dimension n (%)^a^								
no problems^c^	596 (31.2)	83 (36.8)	48 (27.9)	76 (29.9)	94 (27.7)	64 (41.3)	231 (39.1)	
moderate problems^d^	897 (60.0)	86 (49.9)	110 (65.9)	152 (63.5)	191 (61.4)	83 (47.9)	275 (50.1)	
extreme problems^e^	166 (8.7)	25 (13.2)	10 (6.2)	16 (6.6)	32 (11.0)	17 (10.8)	66 (10.8)	0.0006^b^
								
*EQ VAS score*								
Mean (SD)	64.3 (22.9)	70.6 (20.3)	64.1 (22.7)	60.6 (21.4)	60.2 (23.5)	72.0 (19.1)	63.8 (24.2)	
Median (25%-75% quantile)	70 (50 - 80)	77.5 (60 - 80)	70 (50 - 80)	60 (50 - 75)	60 (50 - 80)	75 (60 - 85)	70 (50 - 80)	
Weighted mean (SE)	61.9 (0.74)	69.3 (1.61)	62.0 (2.29)	60.5 (1.47)	60.0 (1.22)	72.9 (1.91)	62.5 (1.33)	< 0.0001^f^
								
*PCS score*								
Mean (SD)	41.8 (10.9)	42.5 (11.2)	42.6 (10.1)	39.6 (10.9)	41.4 (11.0)	40.9 (10.8)	42.7 (10.8)	
Median (25%-75% quantile)	43.6 (34.2 - 51.0)	44.6 (35.2 - 51.8)	44.7 (35.9 - 50.8)	40.8 (31.1 - 48.2)	43.7 (31.8 - 50.3)	41.7 (33.0 - 50.1)	44.3 (36.1 - 51.8)	
Weighted mean (SE)	41.1 (0.34)	42.1 (0.98)	43.0 (0.76)	39.6 (0.70)	40.9 (0.70)	41.2 (1.06)	42.0 (0.55)	0.0407^f^
								
*MCS score*								
Mean (SD)	54.3 (8.7)	57.2 (7.8)	54.4 (8.5)	56.3 (7.3)	52.5 (9.1)	56.1 (7.4)	52.8 (9.3)	
Median (25%-75% quantile)	56.8 (50.5 - 60.6)	59.3 (55.3 - 61.8)	56.5 (49.9 - 60.6)	58.0 (53.0 - 60.8)	55.0 (48.9 - 58.8)	57.9 (53.2 - 60.8)	55.4 (47.8 - 59.8)	
Weighted mean (SE)	54.3 (0.25)	56.7 (0.66)	54.1 (0.64)	56.4 (0.50)	52.2 (0.46)	55.9 (0.72)	52.4 (0.50)	< 0.0001^f^

^a ^Raw numbers (weighted proportions); ^b ^weighted chi-square test for differences between countries; ^c ^no problems in any dimension; ^d ^moderate problems in at least one dimension but no extreme problems in any dimension; ^e ^extreme problems in at least one dimension; ^f ^weighted regression analysis testing for deviation of country specific means from grand mean.

The weighted mean EQ VAS score was 61.9 for the total sample, ranging from 60.0 in Italy to 72.9 in the Netherlands (p < 0.0001). The total sample's weighted mean SF-12 PCS score was 41.1, ranging from 39.6 in Germany to 43.0 in France (p = 0.0407); and the weighted mean SF-12 MCS score was 54.3, ranging from 52.2 in Italy to 56.7 in Belgium (p < 0.0001). Thus, the countries significantly differed regarding the weighted mean EQ VAS and SF-12 derived MCS score, but not the PCS score. Weighting had little impact on any of the three summary scores though; the difference between weighted and unweighted scores was always below 2.5.

When dissecting the total sample by age-groups (Table 3), the proportion of respondents with no problems in any one of the dimensions significantly decreased with age, from 34.6% for the age-group 75-79 to 18.9% for respondents aged 85+ (p = 0.0013). Age groups moreover differed significantly within EQ-5D dimensions mobility (p < 0.0001), self-care (p < 0.0001), and usual activities (p < 0.0001), i.e. the proportion of respondents stating "no problems" significantly decreased. The weighted mean EQ-VAS score significantly decreased with age as well, from 64.1 for the age-group 75-79 to 56.7 for respondents aged 85+ (p = 0.0006). Similarly, the weighted mean PCS score decreased from 42.3 to 37.9 (p < 0.0001). However, the age groups did not differ regarding the MCS score (p = 0.5331).

**Table 3:** Problems in EQ-5D dimensions, EQ VAS, PCS and MCS scores, by age groups

	Age 75-79 n = 945	Age 80-84 n = 461	Age 85+ n = 253	p-value
*EQ-5D dimension*				
Mobility n (%)^a^				
no problems	557 (54.4)	254 (50.4)	95 (32.2)	
moderate problems	379 (44.5)	202 (48.8)	153 (66.4)	
extreme problems	9 (1.1)	5 (0.8)	5 (1.4)	< 0.0001^b^
Self care n (%)^a^				
no problems	829 (87.5)	381 (80.7)	174 (62.0)	
moderate problems	96 (10.5)	74 (18.2)	69 (32.5)	
extreme problems	20 (2.0)	6 (1.0)	10 (5.4)	< 0.0001^b^
Usual activities n (%)^a^				
no problems	667 (70.2)	292 (61.9)	116 (39.5)	
moderate problems	240 (26.4)	143 (34.0)	113 (50.8)	
extreme problems	38 (3.4)	26 (4.1)	24 (9.7)	< 0.0001^b^
Pain/discomfort n (%)^a^				
no problems	493 (46.6)	233 (44.5)	102 (38.3)	
moderate problems	402 (48.8)	203 (50.4)	137 (54.3)	
extreme problems	50 (4.6)	25 (5.2)	14 (7.4)	0.2981^b^
Anxiety/depression n (%)^a^				
no problems	843 (89.0)	415 (87.1)	226 (88.3)	
moderate problems	89 (10.0)	43 (12.1)	25 (11.2)	
extreme problems	13 (1.0)	3 (0.8)	2 (0.6)	0.8748^b^
Any dimension n (%)^a^				
no problems^c^	379 (34.6)	164 (30.8)	53 (18.9)	
moderate problems^d^	478 (57.6)	253 (61.1)	166 (67.7)	
extreme problems^e^	88 (7.8)	44 (8.1)	34 (13.4)	0.0013^b^
				
*EQ VAS score*				
Mean (SD)	65.7 (22.1)	63.3 (23.1)	60.5 (25.3)	
Median (25%-75% quantile)	70 (50 - 80)	70 (50 - 80)	65 (50 - 80)	
Weighted mean (SE)	64.1 (0.90)	59.8 (1.42)	56.7 (1.47)	0.0006^f^
				
*PCS score*				
Mean (SD)	42.8 (10.8)	41.2 (10.9)	39.0 (10.5)	
Median (25%-75% quantile)	44.8 (35.5 - 51.9)	43.1 (34.0 - 50.3)	40.2 (14.6 - 61.3)	
Weighted mean (SE)	42.3 (0.42)	40.2 (0.57)	37.9 (0.68)	< 0.0001^f^
				
*MCS score*				
Mean (SD)	54.0 (9.0)	54.7 (8.4)	54.5 (8.2)	
Median (25%-75% quantile)	56.9 (50.2 - 60.6)	57.0 (51.1 - 60.7)	56.1 (49.5 - 60.7)	
Weighted mean (SE)	54.0 (0.34)	54.9 (0.39)	54.1 (0.52)	0.5331^f^

^a ^Raw numbers (weighted proportions); ^b ^weighted chi-square test for differences between age-groups; ^c ^no problems in any dimension; ^d ^moderate problems in at least one dimension but no extreme problems in any dimension; ^e ^extreme problems in at least one dimension; ^f ^weighted regression analysis testing for deviation of age-group specific means from grand mean.

### *Multivariate *analysis of problems in EQ-5D dimensions

To further examine the impact of country and socio-demographic variables on EQ-5D dimensions, a multiple logistic regression analysis was performed (Table 4). Similar to findings from bivariate analyses, age was a significant predictor of problems in EQ-5D dimensions mobility, self care, and usual activities, but also of problems in the dimension pain/discomfort. Female gender was associated with more problems in three out of five dimensions. Consequently, older respondents and females were also more likely to report problems in at least one of the dimensions ("any dimension"). Short duration of education and low income were each associated with more problems in one ED-5 D dimension, i.e. self care and pain/discomfort, respectively. Respondents from Italy reported problems in three of the dimensions (usual activities, pain/discomfort, anxiety/depression) significantly more frequently than the grand mean and elders from France in one dimension (anxiety/depression), whereas respondents from Spain tended to report problems in the dimension pain/discomfort significantly less frequently than the grand mean.

**Table 4:** Results of weighted logistic regression with problems in EQ-5D dimensions used as dependent variables (n = 1659)

Independent variable	Problems in dimension mobility		Problems in dimension self care		Problems in dimension usual activities		Problems in dimension pain/discomfort		Problems in dimension anxiety/depression		Problems in any dimension	
	OR	99% CI	OR	99% CI	OR	99% CI	OR	99% CI	OR	99% CI	OR	99% CI
Age (years)	1.10**	1.06 - 1.15	1.15**	1.10 - 1.20	1.12**	1.07 - 1.17	1.05*	1.01 - 1.09	1.00	0.94 - 1.07	1.08**	1.04 - 1.13
Male gender (ref. female)	0.51**	0.35 - 0.75	0.69	0.42 - 1.10	0.49**	0.32 - 0.73	0.49**	0.33 - 0.71	0.55	0.30 - 1.02	0.57**	0.38 - 0.85
Education > 12 years (ref. education ≤ 12 years)	0.91	0.53 - 1.56	0.45*	0.23 - 0.88	0.83	0.47 - 1.44	0.90	0.53 - 1.54	0.74	0.34 - 1.59	0.97	0.56 - 1.68
Paid employment (ref. no paid employment)	0.99	0.49 - 1.98	1.35	0.63 - 2.90	1.09	0.54 - 2.20	0.71	0.33 - 1.52	0.74	0.22 - 2.47	0.88	0.42 - 1.86
Income > median (ref. income ≤ media)	0.70	0.49 - 1.02	0.85	0.55 - 1.33	0.95	0.66 - 1.36	0.69*	0.49 - 0.98	0.82	0.49 - 1.39	0.73	0.50 - 1.06
Living with partner (ref. not living with partner)	1.15	0.78 - 1.68	0.93	0.56 - 1.56	1.09	0.71 - 1.67	1.04	0.71 - 1.52	1.35	0.74 - 2.46	0.94	0.63 - 1.41
Belgium^a ^	0.90	0.60 - 1.36	1.12	0.66 - 1.90	1.12	0.81 - 1.55	0.91	0.61 - 1.34	0.65	0.33 - 1.32	0.91	0.63 - 1.33
France^a^	1.02	0.70 - 1.49	1.04	0.70 - 1.53	0.92	0.63 - 1.33	1.44	0.95 - 2.19	1.85*	1.04 - 3.28	1.31	0.82 - 2.09
Germany^a^	1.40	0.99 - 1.96	0.95	0.61 - 1.49	0.97	0.68 - 1.37	0.94	0.67 - 1.31	0.70	0.36 - 1.39	1.20	0.83 - 1.72
Italy^a^	1.22	0.89 - 1.67	1.35	0.90 - 2.03	1.49**	1.09 - 2.03	1.50*	1.09 - 2.06	2.09**	1.33 - 3.27	1.29	0.92 - 1.82
Netherlands^a^	0.65	0.42 - 1.01	0.68	0.34 - 1.34	0.66	0.41 - 1.05	0.80	0.53 - 1.22	0.38	0.14 - 1.05	0.70	0.46 - 1.09
Spain^a^	0.98	0.73 - 1.32	0.99	0.66 - 1.50	1.03	0.77 - 1.39	0.68**	0.51 - 0.91	1.46	0.92 - 2.24	0.77	0.57 - 1.04

OR, odds ratio; CI, confidence interval; ^a ^effect coding for deviation of country-specific mean from grand mean; * p < 0.01; ** p < 0.001

Multiple linear regression analysis revealed that respondents from Germany and Italy rated their health state significantly lower on the EQ-VAS, while those from Belgium and the Netherlands tended to rate it significantly higher than the grand mean, even when controlling for socio-demographic variables (Table 5 Model 1). Since EQ-VAS scores decreased with old age, these results support the findings from Table 3. EQ-VAS scores were also higher for persons which had received more than 12 years of education.

**Table 5:** Results of weighted ordinary least square regression models with EQ VAS score used as dependent variable (n = 1659)

Independent variable	**Model 1 (R**^**2 **^**= 0.06)**			**Model 2 (R**^**2 **^**= 0.31)**		
	Coefficient value	Standard error	p-value	Coefficient value	Standard error	p-value
Intercept	62.54	1.24	< 0.0001	76.73	1.36	< 0.0001
Age (years, centred)	-0.74	0.18	< 0.0001	-0.11	0.16	0.4874
Male gender (ref. female)	2.77	1.80	0.1242	-1.56	1.69	0.3561
Education > 12 years (ref. education ≤ 12 years)	6.82	2.17	0.0017	5.24	2.22	0.0186
Paid employment (ref. no paid employment)	-3.23	3.87	0.4043	-3.43	3.53	0.3308
Income > median (ref. income ≤ media)	3.34	1.55	0.0320	1.67	1.36	0.2203
Living with partner (ref. not living with partner)	-3.34	1.88	0.0759	-2.63	1.64	0.1105
Belgium^a^	4.42	1.68	0.0086	3.90	1.40	0.0054
France^a^	-2.77	2.14	0.1957	-1.97	2.02	0.3303
Germany^a^	-4.20	1.34	0.0018	-4.01	1.14	0.0004
Italy^a^	-3.66	1.33	0.0059	-1.13	1.13	0.3189
Netherlands^a^	7.70	1.74	< 0.0001	4.78	1.31	0.0003
Spain^a^	-1.50	1.38	0.2799	-1.58	1.15	0.1698
Problems mobility (ref. no problems mobility)	-	-	-	-10.53	1.61	< 0.0001
Problems self-care (ref. no problems self-care)	-	-	-	-8.90	2.15	< 0.0001
Problems usual activities (ref. no problems usual activities)	-	-	-	-6.79	1.63	< 0.0001
Problems pain/discomfort (ref. no problems pain/discomfort)	-	-	-	-3.38	1.58	0.0329
Problems anxiety/depression (ref. no problems anxiety/depression)	-	-	-	-11.31	2.50	< 0.0001

^a ^effect coding for deviation of country-specific mean from grand mean

In order to identify differences in the valuation of identical EQ-5D health states on the EQ-VAS, an additional regression analysis was preformed which included response levels of each EQ-5D dimension as independent variables (Table 5 Model 2). Including the EQ-5D response levels considerably increased the model fit (R^2 ^= 0.31) on the one hand, while independent variables age and income became insignificant on the other. Thus, worse EQ-VAS scores for the older of these elderly respondents are explained by their worse health state, i.e. more problems reported in each of the EQ-5D dimensions, but not by age itself. Differences between countries remained, with the exception of Italy and France. Results show that after controlling for health state and socio-demographic variables, respondents from Germany still rated their health significantly worse, while those from Belgium and the Netherlands reported significantly higher EQ-VAS scores than the grand mean. None of the socio-demographic variables included in the second model significantly affected the EQ-VAS score. As expected, problems within the EQ-5D dimensions had the strongest negative association with the EQ-VAS score, in particular anxiety/depression and mobility. Yet, the impact of the dimension "pain/discomfort" in the EQ-VAS score did not reach the level of significance (p = 0.0329).

Similar to the analysis above, we next used SF-12 derived PCS and MCS scores each as a dependent variable in a weighted ordinary least square regression, while again controlling for socio-demographic variables (Table 6). It was found that age and German residence were negatively associated with the PCS score, while for male gender and higher education (> 12 years) a positive association was observed. Male gender also had a positive effect on the MCS score, as did residence in Belgium and Germany. Living in Italy and Spain on the other hand was associated with lower than grand mean MCS scores (p < 0.0001). Interestingly, German respondents rated their mental health 1.84 MCS points above the grand mean, but their physical health 1.86 PCS points below the grand mean, irrespective of age, education, income, living situation, and employment status. Although each model explains very little of the observed variance, i.e. R^2 ^= 0.08 for the PCS model and R^2 ^= 0.06 for the MCS model, these findings nonetheless underscore and augment in the findings from Table 2.

**Table 6:** Results of weighted ordinary least square regression models with PCS and MCS scores used as dependent variable (n = 1659)

Independent variable	**Model 1 (R**^**2 **^**= 0.06)**			**Model 2 (R**^**2 **^**= 0.31)**		
	Coefficient value	Standard error	p-value	Coefficient value	Standard error	p-value
Intercept	39.64	0.59	< 0.0001	53.49	0.47	< 0.0001
Age (years, centred)	-0.45	0.07	< 0.0001	0.05	0.06	0.4180
Male gender (ref. female)	2.67	0.74	0.0003	1.83	0.58	0.0017
Education > 12 years (ref. education ≤ 12 years)	2.95	0.95	0.0019	0.82	0.67	0.2214
Paid employment (ref. no paid employment)	-0.06	1.63	0.9690	-0.78	1.14	0.4921
Income > median (ref. income ≤ media)	1.18	0.71	0.0974	1.06	0.60	0.0760
Living with partner (ref. not living with partner)	-0.56	0.75	0.4615	-0.38	0.65	0.5576
Belgium^a^	0.31	0.84	0.7122	2.02	0.74	0.0069
France^a^	1.42	0.88	0.1053	-0.52	0.67	0.4356
Germany^a^	-1.86	0.65	0.0043	1.84	0.48	0.0001
Italy^a^	-0.24	0.68	0.7255	-2.32	0.49	< 0.0001
Netherlands^a^	-0.50	0.91	0.5816	1.26	0.64	0.0489
Spain^a^	0.88	0.58	0.1304	-2.27	0.50	< 0.0001
R^2^	0.08			0.06		

^a ^effect coding for deviation of country-specific mean from grand mean

## Discussion

The purpose of this study was to evaluate and compare self-reported health status of the advanced elderly population in six European countries via EQ-5D and SF-12. More than two thirds of all respondents reported problems in at least one of the EQ-5D dimensions with problems in the dimension pain/discomfort being most frequent and problems in the dimension anxiety/depression being least frequent. Extreme problems in any of the EQ-5D dimensions were indicated by 8.7%. These proportions are much higher than in the rest of the ESEMeD sample aged below 75 years of whom only about one third (32.2%) reported problems in one or more of the EQ-5D dimensions, with only 2.4% reporting extreme problems (data not shown). The mean EQ-VAS score of 61.9 points at substantially reduced valuation of health status by the advanced elderly respondents as compared to the mean score of 78.4 reported by respondents younger than 75 years. While the mean PCS score of the advanced elderly was well below the mean of the general population norm, indicating markedly impaired physical health status, the mean MCS score was similar to the general population norm, indicating that mental health status was not worse than in the rest of the population.

Even within the group of advanced elderly, the frequency of problems strongly increased with age, with more than 80% of respondents aged 85+ reporting problems in at least one of the EQ-5D dimensions compared to 65% of those aged 75-79; noteworthy, the proportion of respondents reporting extreme problems increased even more strongly, being 1.7 times higher in respondents aged 85+ than in those aged 75-79. The valuation of health status on the EQ-VAS and physical health status measured by the PCS also decreased significantly with age, whereas mental health status measured by the MCS was not associated with age.

Female gender was associated with more problems in most of the EQ-5D dimensions, lower PCS and MCS scores, even when controlling for other sociodemographic variables. The gender difference was most pronounced in the oldest age group (85+) where, e.g., 86% of female respondents reported problems in at least one of the EQ-5D dimensions compared to only 70% of male respondents. However, valuation of health status on the EQ-VAS was not significantly different between genders. The finding that women were more likely to report problems in EQ-5D dimensions as well as lower PCS and MCS scores was also observed in previous studies [9,20]. Research concerned with the total adult population (18 and older) similarly found significant gender differences; i.e. females typically reported more problems in EQ-5D [10,14,20] dimensions and lower PCS and MCS scores [21]. In comparison to studies that solely analyzed elderly cohorts the differences were much less pronounced though. Since none of the studies investigating total adult populations controlled for age, it remains uncertain if the observed differences may not be the result of pronounced differences in the older age groups.

When analysing the total sample by country, significant differences between the countries surfaced, especially in EQ-5D dimensions usual activities, anxiety/depression, and pain/discomfort, with the proportion of respondents in Italy reporting problems in at least one of the EQ-5D dimension being 1.23 times higher than in the Netherlands. Multivariate analysis confirmed that especially Italian respondents reported significantly more problems. The valuation of health status on the EQ-VAS differed between countries as well. While respondents from the Netherlands and Belgium tended to rate health states high, elders from Germany rated them low. Even when controlling for socio-economic variables and health problems, a difference of around nine (8.79) in mean EQ-VAS scores occurred between respondents from the Netherlands and Germany, which can be considered meaningful from a clinical point of view [22]. Similarly, countries differed in reported MCS, and to a lesser extent PCS scores. In cross-country studies it is always difficult to differentiate between 'real' differences in health states vs. differences that may be due to different response styles, different reference levels or differences in the meaning of response levels within the various language versions of the questionnaires. According to Jurges [23] large cross-country variations in self-reported general health are only partly reflected by 'true' differences in health states. Therefore a possible reason for the Italian respondent being depressed more frequently, and German respondents rating their health worse than respondents from other countries may be cultural differences in values and reference levels. On the other hand, a possible reason for 'true' differences in health between these non-institutionalized country samples may be country-specific differences in the probability of being admitted to a nursing home when the health states deteriorates.

Other general population surveys using the EQ-5D in the elderly tended to report even higher proportions of respondents with problems in the various EQ-5D dimensions. E.g., in a survey of 190 elders aged 75+ conducted in the USA, Johnson et al. [14] reported higher problem frequencies for the EQ-5D dimensions mobility (59.5%), usual activities (52.4%), pain/discomfort (70.1%) and, in particular, anxiety/depression (36.3%) than in any of our country samples; problems in self-care (18.5%) and EQ-VAS score (67.7) were similar though. For a general population sample of 173 individuals aged 75+ from Italy, Savioa et al. [20] reported higher problem frequencies in pain/discomfort (67.3%) and anxiety/depression (53.3%), similar frequencies in usual activities (37.3%) and self-care (21.8%), and a lower frequency in mobility (40.0%); the EQ-VAS score was 68.0. Surveys which used the SF-12 in the elderly population samples also reported impaired physical health but average mental health summary scores [20,21,24], similar to our survey. Yet, similar to our results, mean MCS scores reported by these surveys did not differ from general population average, indicating that mental health does not deteriorate with age.

The main limitation of the current analysis is due to the fact that the sample consists solely of non-institutionalized elderly persons. Hence, the results do not apply to older institutionalized individuals, i.e. those being hospitalized or living in care facilities such as nursing homes. Since institutionalization is associated with age one the one hand (is more frequent within the advanced elderly), and institutionalized persons considerably differ from non-institutionalized elders in many aspects on the other (e.g. their health is more seriously impaired), excluding this subgroup may have biased our results towards an overestimation of the actual health status within this population group.

The results of this survey point at substantially impaired health status of the elderly population, especially of the advanced elderly. As this is the fastest growing population, health care systems throughout Europe will face a tremendous challenge in trying to meet the health care needs of this population group. The elderly subjects included in our analysis may however exhibit worse health states than the baby booming cohort will when reaching the same age in the future. Recent supportive evidence of the "Compression of Morbidity" paradigm [25] points to a later onset of many diseases and disability, which has far exceeded the rise in life expectancy during the same period [26-29]. Younger cohorts in developed countries tend to live longer, whereas the time of serious morbidity and especially disability is increasingly getting compressed into the last years of life [30-32]. Somewhat contradictory, the prevalence of middle aged and especially elderly persons diagnosed with (and treated for) multiple chronic conditions has substantially increased at the same time [33,34], and multiple chronic conditions are associated with substantial excess health care expenditures [35,36]. Due to better medical treatment these patients may be suffering from disability to a lower degree. Even if the rate of functional decline will keep on dropping by some percentage points into the future, the impact of demographic change (i.e. the numerical increase in the size of the aged population over the next 30 years) will lead to even larger numbers of people in need of complex care. Appropriate and effective medical treatment for this growing group of patients will most likely require additional health care resources.

## Competing interests

The authors declare that they have no competing interests.

## Authors' contributions

HHK, MCA, GV, RB, JMH, GdG, RdG, and VK designed the study and managed the field work. HHK, DH, HM undertook the statistical analyses. HHK, TL, and SRH wrote the first draft of the manuscript. All authors contributed to and have approved the final manuscript.
